# Supplementary material for: Differential co-expression network analysis with DCoNA reveals isomiR targeting aberrations in prostate cancer
Source: Bioinformatics. 2023 Jan 23;39(2):btad051. doi: 10.1093/bioinformatics/btad051 (PMC9901399; doi:10.1093/bioinformatics/btad051)
Supplement: btad051_Supplementary_Data [file btad051_supplementary_data.pdf]

Supplementary materials

IsomiRs			Targets		
**IsomiR**	$\log_2$(**fold change**)	**FDR**	**Initial**	**Disappeared**	**Appeared**
hsa-miR-28-3p|0	-0.00	1.00	6	1	0
hsa-let-7b-5p|0	-0.13	0.23	44	19	4
hsa-miR-30a-3p|0	-0.36	0.01	29	14	0
hsa-miR-101-3p|-1	-0.21	0.01	38	11	0
hsa-let-7c-5p|0	-0.34	< 0.01	20	3	0
hsa-miR-30e-3p|0	-0.33	< 0.01	33	12	0
hsa-miR-145-5p|0	-0.44	< 0.01	53	5	0
hsa-miR-99b-5p|0	-0.34	< 0.01	1	0	0
hsa-miR-22-3p|0	-0.35	< 0.01	14	4	0
hsa-miR-29c-3p|0	-0.50	< 0.01	27	6	0
hsa-miR-99a-5p|0	-0.63	< 0.01	2	0	0
hsa-miR-10a-5p|0	-0.99	< 0.01	16	6	0
hsa-miR-101-3p|0	-0.67	< 0.01	19	4	0
hsa-miR-29a-3p|0	-0.68	< 0.01	33	12	0
hsa-miR-30e-5p|0	-0.59	< 0.01	59	19	0
hsa-miR-143-3p|-1	-1.21	< 0.01	11	1	0
hsa-miR-26a-5p|0	-0.71	< 0.01	49	11	1
hsa-miR-143-3p|0	-1.37	< 0.01	24	6	0
hsa-miR-27b-3p|0	-1.10	< 0.01	77	16	0
hsa-miR-10b-5p|+1	0.23	0.18	8	3	0
hsa-miR-30a-5p|0	0.17	0.17	63	13	1
hsa-miR-10b-5p|0	0.55	< 0.01	12	5	0
hsa-let-7f-5p|0	0.47	< 0.01	35	9	0
hsa-let-7a-5p|0	0.46	< 0.01	34	10	2
hsa-miR-30d-5p|0	0.49	< 0.01	69	28	4
hsa-miR-21-5p|0	0.62	< 0.01	7	2	1
hsa-miR-375|+1	1.26	< 0.01	0	0	0
hsa-miR-103a-3p|0	0.81	< 0.01	18	1	0
hsa-miR-183-5p|0	1.66	< 0.01	42	16	1
hsa-miR-92a-3p|0	1.09	< 0.01	26	4	7
hsa-miR-25-3p|0	1.02	< 0.01	37	7	1
hsa-miR-183-5p|+1	1.96	< 0.01	50	16	1
hsa-miR-375|0	1.88	< 0.01	12	4	0
hsa-miR-148a-3p|0	1.44	< 0.01	67	17	0
hsa-miR-182-5p|0	1.93	< 0.01	108	27	0
hsa-miR-200c-3p|0	1.58	< 0.01	151	46	2
hsa-miR-93-5p|0	1.77	< 0.01	29	3	20

Table 1: **IsomiRs**: ifferential expression of the highly expressed isomiRs in TCGA-PRAD ("Normal" vs "Tumor"). **Initial**: the number of bioinformatically predicted mRNA targets in "Normal" state with correlation < -0.3. **Disappeared**: the number of targets significantly "jumped" over the correlation threshold and lost negative correlation. **Appeared**: the number of targets significantly "jumped" over the correlation threshold and acquired negative correlation.

Risk group	Control	Low	Intermediate	High
n	5	40	16	91
Age, years (median, [$p25, p75$])	32 [30, 33]	62.5 [58, 68]	63.5 [58, 67]	65.5 [61, 69]
Preoperative level of PSA, ng/ml		8 [6.3, 10.4]	10 [6.5, 19.9]	11.8 [5.3, 27.7]
**TNM classification**				
T1		2		
T2		38	16	17
T3				54
T4				20
N0		40	16	57
N1				34
M0		40	16	60
M1				31
**Gleason score**				
4		1		
5				5
6		39		9
7			16	22
8				35
9				14
10				6

Table 2: Clinical characteristics of patients included in the study.

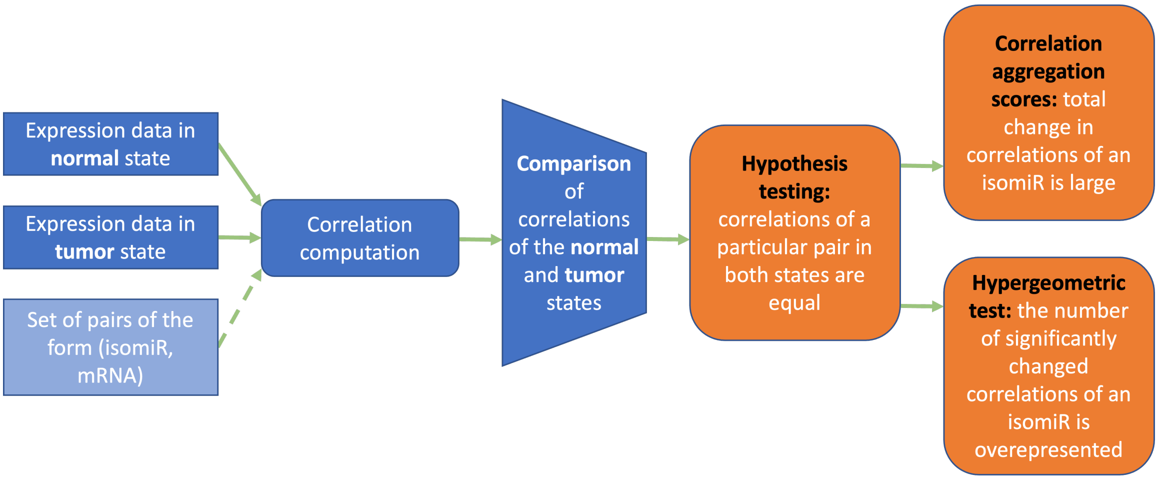

Figure 1: Computational pipeline of DCoNA. The dashed line represents an optional network mode. If the set of pairs of interest is not defined, DCoNA performs the differential co-expression analysis for all possible pairs of molecules from the expression data.

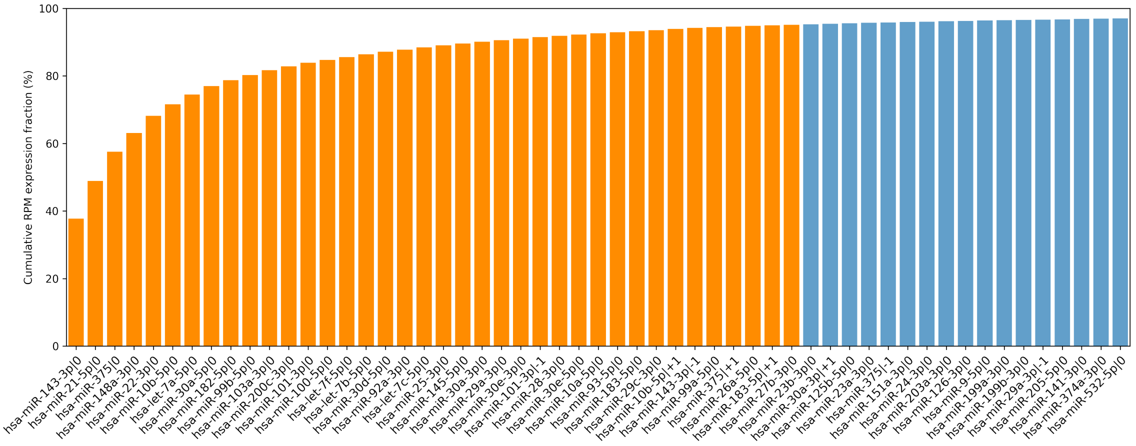

Figure 2: Cumulative expression fraction of isomiRs in TCGA-PRAD dataset. The orange bars define 95% threshold, i.e. 95% of expression comes from 38 isomiRs. IsomiRs are sorted in descending order of expression.

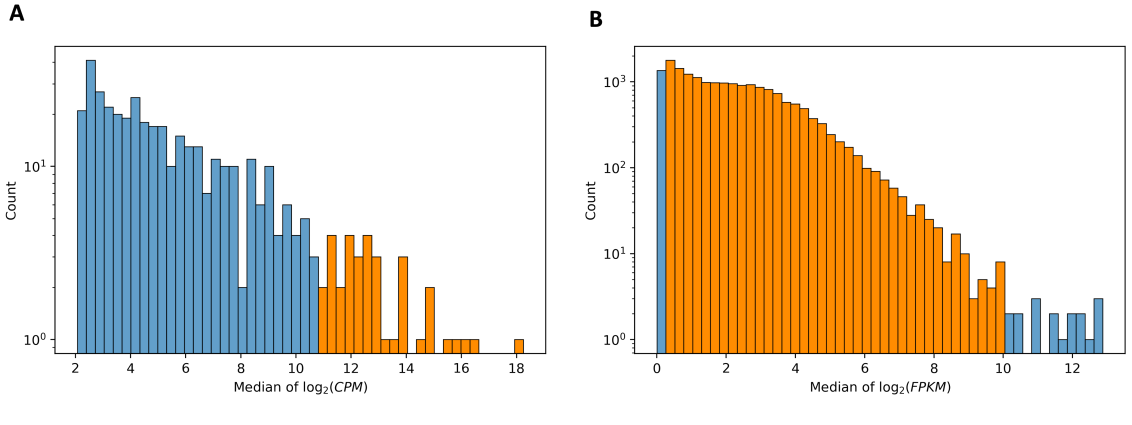

Figure 3: Histograms of **A**: isomiRs and **B**: mRNAs expression in TCGA-PRAD. The orange bars higlight the analised molecules: the higly expressed isomiRs and their target mRNAs (bionfirmatically predicted and validated with correlation).

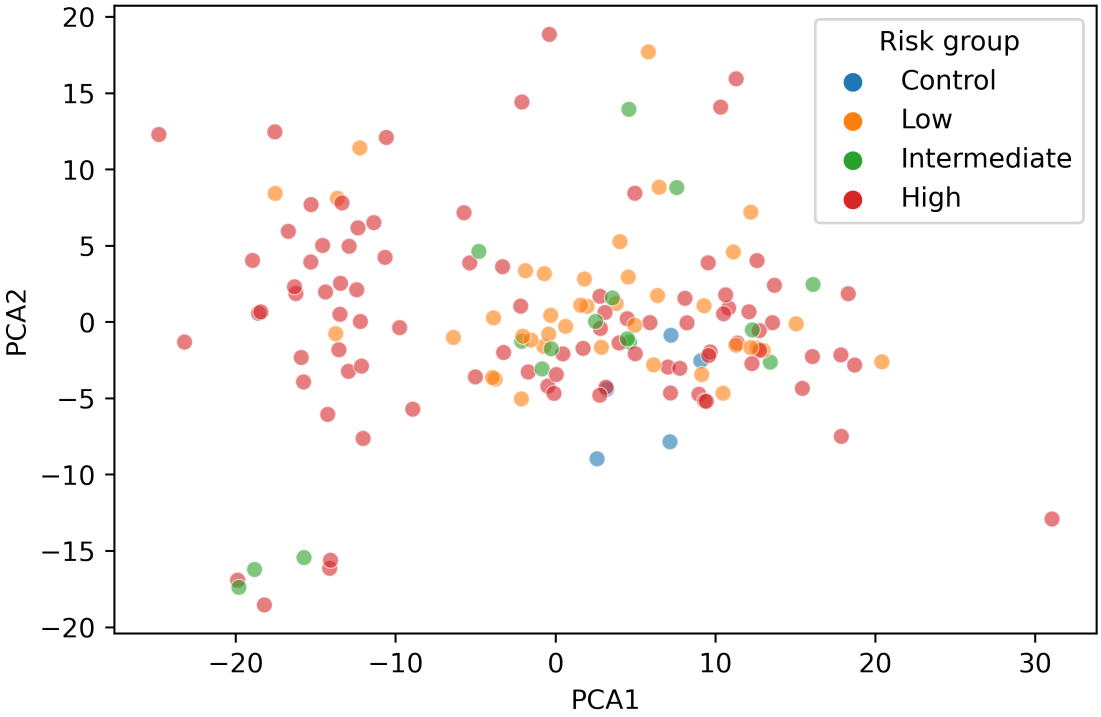

Figure 4: PCA of miRNA expression in the microarray dataset. The first two PCA coordinates account for 25% of the variance.

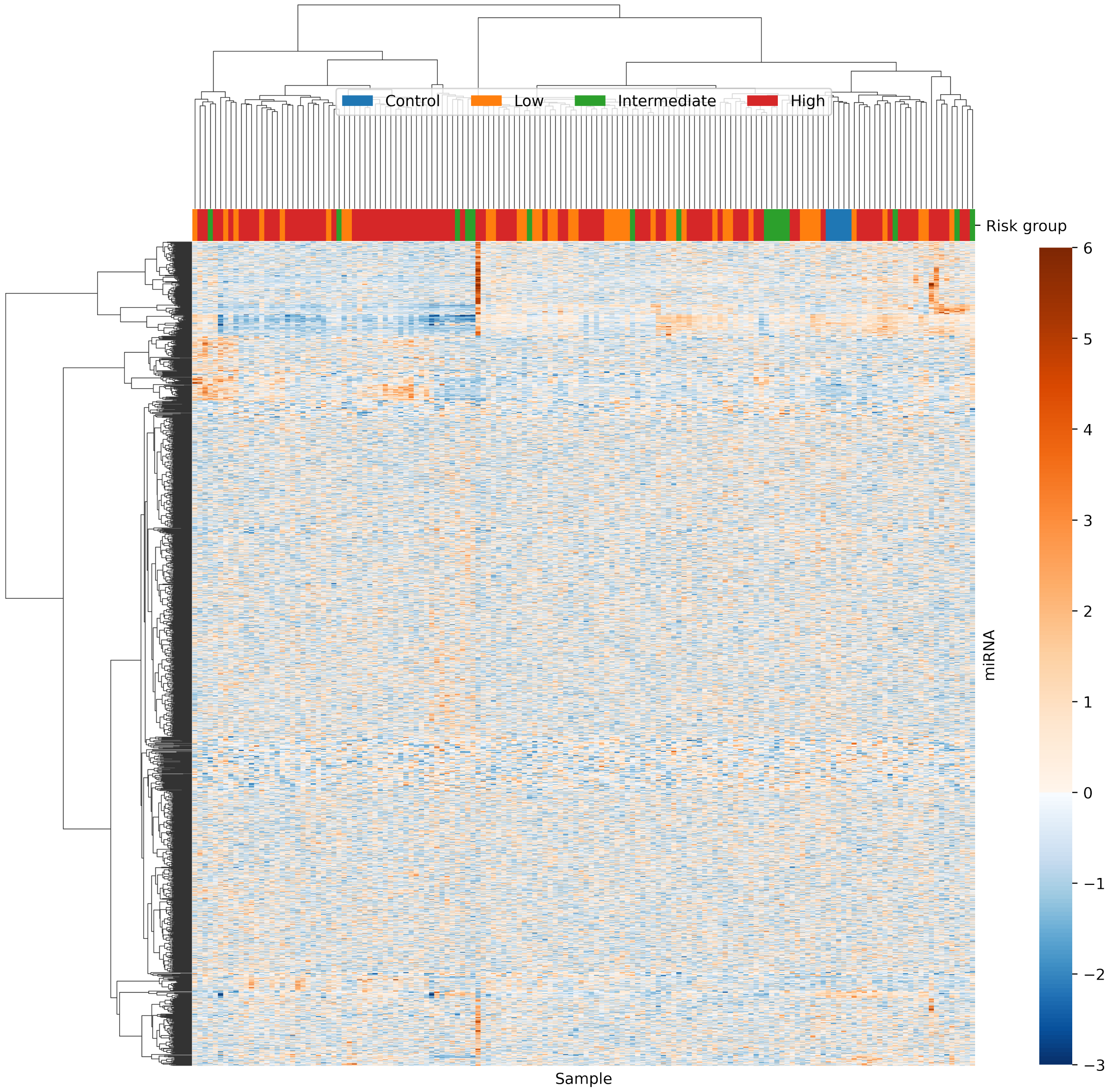

Figure 5: Clustermap of miRNA expression in the microarray dataset. MiRNA expression of every sample was z-transformed before clustering.
